# Quantitative Proteomic Profiling of Tachyplesin I Targets in U251 Gliomaspheres

**DOI:** 10.3390/md15010020

**Published:** 2017-01-18

**Authors:** Xuan Li, Jianguo Dai, Yongjun Tang, Lulu Li, Gang Jin

**Affiliations:** School of Applied Chemistry and Biotechnology, Shenzhen Polytechnic, No. 2190 Liuxian Road, Nanshan District, Shenzhen 518055, Guangdong, China; smilelixuan@163.com (X.L.); jgdai@szpt.edu.cn (J.D.); tangyongjun@szpt.edu.cn (Y.T.); lilulu011071@hotmail.com (L.L.)

**Keywords:** tachyplesin I, glioblastoma multiforme, cancer stem cell, stable isotope dimethyl labeling, parallel reaction monitoring

## Abstract

Tachyplesin I is a cationic peptide isolated from hemocytes of the horseshoe crab and its anti-tumor activity has been demonstrated in several tumor cells. However, there is limited information providing the global effects and mechanisms of tachyplesin I on glioblastoma multiforme (GBM). Here, by using two complementary proteomic strategies (2D-DIGE and dimethyl isotope labeling-based shotgun proteomics), we explored the effect of tachyplesin I on the proteome of gliomaspheres, a three-dimensional growth model formed by a GBM cell line U251. In total, the expression levels of 192 proteins were found to be significantly altered by tachyplesin I treatment. Gene ontology (GO) analysis revealed that many of them were cytoskeleton proteins and lysosomal acid hydrolases, and the mostly altered biological process was related to cellular metabolism, especially glycolysis. Moreover, we built protein–protein interaction network of these proteins and suggested the important role of DNA topoisomerase 2-alpha (TOP2A) in the signal-transduction cascade of tachyplesin I. In conclusion, we propose that tachyplesin I might down-regulate cathepsins in lysosomes and up-regulate TOP2A to inhibit migration and promote apoptosis in glioma, thus contribute to its anti-tumor function. Our results suggest tachyplesin I is a potential candidate for treatment of glioma.

## 1. Introduction

Gliomas, the most common group of primary brain tumors, are subcategorized into astrocytomas, oligodendrogliomas and ependymomas. According to World Health Organization (WHO), glioblastoma multiforme (GBM), the most malignant and lethal form of brain tumor in adults, is a grade IV astrocytoma with very high morbidity and mortality. The disease has a very poor prognosis with short median survival, only about 15 months, despite current multimodal treatment including maximal surgical resection if feasible, followed by a combination of radiotherapy and/or chemotherapy [1]. Therefore, it is imperative to present new and more effective therapeutic interventions to better control GBM.

In fact, the short median survival of GBM is largely ascribed to the inevitable tumor recurrence. Recent research has paid more attention to the existence of glioma stem cells (GSCs), which are a subgroup of tumor cells with properties that resemble those of neural stem cells, and are able to drive tumorigenesis and likely contribute to rapid tumor recurrence [2]. These cells were first described more than ten years ago and have been demonstrated with the capability of multi-lineage differentiation, self-renewal and extensive proliferation [3]. In addition, GSCs can endure and even thrive in stressful tumor conditions, including hypoxia, oxidative stress, inflammation, acidic stress, and low glucose [4]. Moreover, their resistance to conventional therapy and promotion of tumor angiogenesis also influence clinical practice [5,6]. Thus, GSCs provide new insight into the strategy in GBM therapy.

Three-dimensional growth model, a growth sphere formed by cancer stem cells under specific culture conditions in vitro, is a more reasonable model for tumor biology and drug screening in vitro studies [7,8]. Likewise, GSCs also have the characteristic of forming spheres and clinical data show that the rates of existence of gliomaspheres were more prominent in high grade malignant gliomas [9]. Previously, we isolated gliomaspheres from U251 glioma cell lines and tried to apply it for drug screening. We found that there were undifferentiated GSCs and differentiated cancer cells with different differentiation degrees in gliomaspheres, which were similar to the growth state of glioma in vivo [10]. Our previous data showed that gliomaspheres express stem cell biomarkers nestin and CD133, which are certain phenotypes of GSC, and tachyplesin I inhibited the viability and proliferation of gliomaspheres dose dependently, by damaging the plasma membrane and inducing differentiation of GSCs [11]. These findings indicate that tachyplesin I is a potential anti-tumor drug which may be used in GBM therapy.

Tachyplesin I, a cationic peptide with 17 residues (NH_2_-K-W-C-F-R-V-C-Y-R-G-I-C-Y-I-R-R-C-R-CONH_2_), was originally isolated from hemocytes of the horseshoe crab (*Tachypleus tridentatus*) [12]. It has the ability of anti-enzymatic hydrolysis due to two disulfide-stabilized β-hairpins [13]. Several studies have demonstrated that tachyplesin I can inhibit the proliferation and affect the differentiation of tumor cells, such as hepatocarcinoma, gastric adenocarcinoma and leukemia [14,15]. This peptide has also been demonstrated to activate the classic complement pathway to lyse and kill tumor cells and to alter the expression of tumor suppressor genes and oncogenes to induce cell differentiation and reverse the malignant phenotype [16,17]. Most interestingly, the negatively charged components of cancer cells, which are quite different from neutral normal cells, are more vulnerable by the positively charged cationic peptides, including tachyplesin I. The electrostatic attraction between cancer cells and cationic peptides is believed to play a major role in the selective disruption of cancer cell membranes, which avoids traditional mechanism of drug resistance [18].

Although the anti-tumor effect of tachyplesin I has been studied to some extent, the mechanism of anti-tumor activity in GBM is largely unknown. In recent years, proteomics has been shown to be a powerful approach for exploring the molecular mechanisms of anti-tumor drugs. In this study, our primary goal was to identify the changes in protein expression profile of U251 gliomaspheres under the treatment of tachyplesin I, which may help us to better understand the molecular mechanisms underlying potential anti-glioma drugs. Here, both gel-based and shotgun proteomic approaches were performed to gain a higher proteome coverage and better quantification results [19]. Proteomic analysis using two dimension difference gel electrophoresis (2D-DIGE) and stable isotope dimethyl labeling based Liquid chromatography–mass spectrometry/mass spectrometry (LC-MS/MS) revealed that 192 proteins were differentially expressed in U251 gliomaspheres in response to tachyplesin I. Biological involvement of these proteins are further discussed in detail through signaling pathways and protein–protein interaction network analysis. Furthermore, the expression of cathepsins in lysosomes and TOP2A was further validated by Western blot and PRM, due to their important involvement in the anti-tumor activity of tachyplesin I, by inhibiting migration and promote apoptosis of glioma cells, respectively.

## 2. Results

### 2.1. Protein Expression Profile of Tachyplesin I Treated U251 Gliomaspheres Using 2D-DIGE Analysis

**Figure 1:**
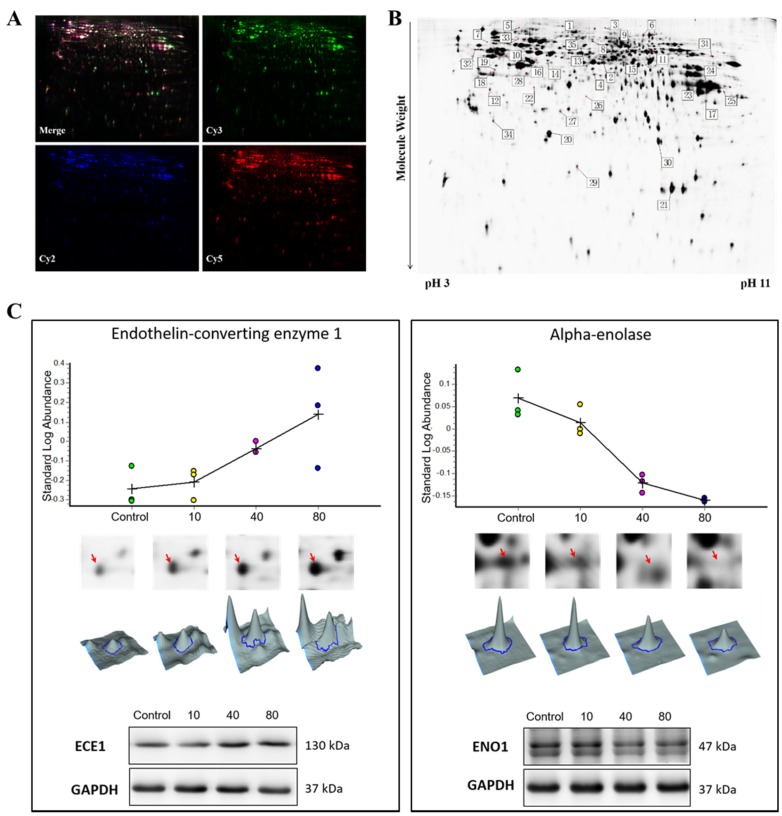
Two dimension difference gel electrophoresis (2D-DIGE) analysis of U251 gliomaspheres after treated with tachyplesin I. (**A**) Representative scanned 2D-DIGE images of Cy2, Cy3, and Cy5, and their overlay derived from a single gel; (**B**) Representative 2D-DIGE protein profiles with the protein spots marked as differentially regulated in U251 gliomaspheres treated with tachyplesin I. Information about the proteins corresponding to the spot numbers is listed in Table 1; (**C**) The expression levels of endothelin-converting enzyme 1 (ECE1) and alpha-enolase (ENO1) in U251 gliomaspheres treated by 0, 10, 40 and 80 μg/mL of tachyplesin I for 24 h are visualized by protein abundance maps (first panel), 2-DE images (second panel), three-dimensional spot images (third panel) and validated by Western blot (bottom panel). GAPDH was used as a loading control.

The 2D-DIGE images, which were scanned at the wavelengths of 488/520, 532/580, and 633/670 nm, visualize the protein expression pattern in the cells (Figure 1A). In the image analysis, 1298 protein spots were detected. Of these, 35 spots with fold change larger than ±1.5 were considered significantly altered in tachyplesin I treated U251 gliomaspheres compared with untreated control (Figure 1B). Among the protein spots that satisfied the statistical criteria, 26 were confidently identified by MALDI-TOF/TOF analysis. Out of 26 identified proteins, 13 were up-regulated while the others were down-regulated in tachyplesin I treated U251 gliomaspheres. Up-regulated proteins were mainly involved in regulation of cell cycle and apoptosis, and cytoskeleton proteins (Table 1). Conversely, down-regulated proteins were involved in glycolysis, response to stimulus and calcium or ion binding (Table 1). Several proteins (Vimentin, Phosphate carrier protein, mitochondrial and Guanine nucleotide-binding protein G(q) subunit alpha) were identified more than once in different location of 2D-DIGE gel, suggesting diverse protein isoforms, such as the occurrence of post-translational modification. Representative images of one up-regulated protein endothelin-converting enzyme 1 (ECE1) and one down-regulated protein alpha-enolase (ENO1) in different dose groups are shown in Figure 1C. Western blot assay was performed to confirm the results obtained from 2D-DIGE experiment and the results were consistent (Figure 1C).

### 2.2. Relative Quantification Using Dimethyl Labeling Based LC-MS/MS Analysis

**Figure 2:**
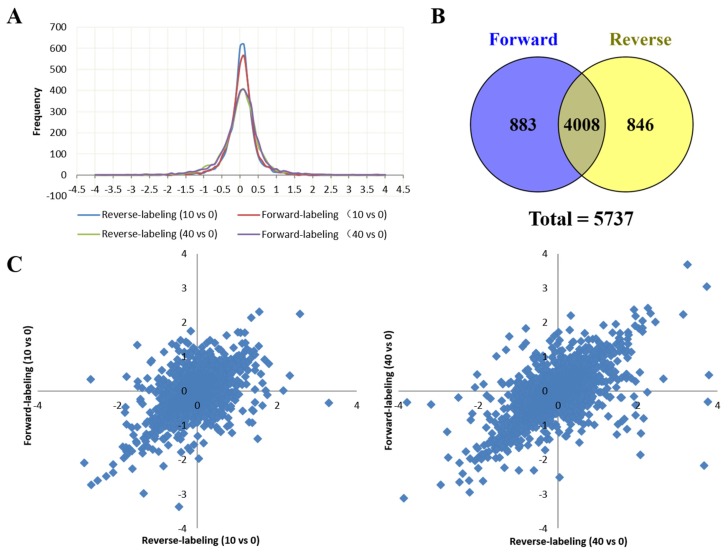

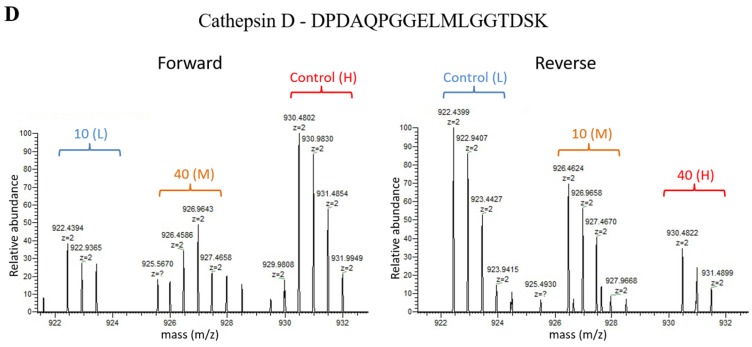
Dimethyl labeling based Liquid chromatography–mass spectrometry/mass spectrometry (LC-MS/MS) analysis of U251 gliomaspheres after treated with tachyplesin I. (**A**) Distribution of quantified protein log_2_ ratios; (**B**) A Venn diagram shows the number of proteins identified in either forward or reverse labeling experiment, as well as the overlap between them; (**C**) A scatter plot showing the forward (*y*-axis) and reverse (*x*-axis) dimethyl labeling log_2_ ratios for the 4008 proteins that were identified and quantified in both experiment, the left panel corresponds to 10 μg/mL group versus control, the right panel corresponds to 40 μg/mL group versus control. The values for each protein are shown as a blue diamond; (**D**) Representative mass spectrometric image revealing the tachyplesin I-induced down regulation of cathepsin D. Shown are the MS for the peptide DPDAQPGGELMLGGTDSK of cathepsin D from the forward (left panel) and reverse (right panel) dimethyl labeling samples.

Peptide samples from the control, and 10 μg/mL and 40 μg/mL tachyplesin I-treated U251 gliomaspheres were labeled with dimethyl stable isotope tags. To obtain reliable quantification results, we conducted one forward and one reverse dimethyl labeling experiments. A total of 74,240 peptides from 4891 proteins were identified in the forward-labeling samples and 73,892 peptides from 4854 proteins in the reverse-labeling samples (Supplementary Materials Tables S1–S4). In both forward and reverse labeling experiment, the labeled peptides account for more than 99.8% of total identified peptides, indicating a good labeling efficiency. A total of 5737 proteins were reliably quantified in both the forward and reverse labeling experiments, of which 4008 proteins were overlapped (Figure 2B). The protein ratios of L/H and M/H in the forward labeling experiment and protein ratios of M/L and H/L in the reverse labeling experiment indicate the relative abundance of proteins in 10 μg/mL and 40 μg/mL tachyplesin I-treated groups compared to the control. The log_2_ transformed protein ratios between two different experimental groups all form a symmetric distribution curve with the peak around zero (the original ratio = 1) (Figure 2A), and proteins that were increased or decreased in the forward-labeling experiment were also increased or decreased in the reverse-labeling experiment (Figure 2C), suggesting that there was no bias in the labeling and LC-MS experiments. Only those proteins with fold changes >2 and quantified in both forward and reverse labeling experiments were reported as differentially expressed proteins. Among 4088 proteins, the expression levels of 166 were significantly altered by tachyplesin I treatment. Among them, 55 were up-regulated (Table 2) while 111 proteins were down-regulated (Table 2). Figure 2D shows representative mass spectrometric results for the identification and quantification of the peptide DPDAQPGGELMLGGTDSK from cathepsin D, which clearly reveals the down-regulation of this protein in both sets of experiments.

### 2.3. Cellular Functions of Differentially Expressed Proteins and Associated Pathways

**Figure 3:**
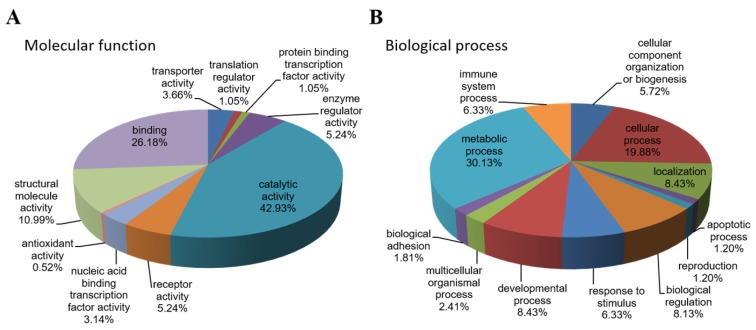
Gene ontology analysis of 192 differentially expressed proteins. The significant (*p* ≤ 0.001) molecular functions (**A**) and biological processes (**B**) are presented in the pie chart.

Systematic gene ontology (GO) analysis of 192 differentially expressed proteins identified from both 2D-DIGE and dimethyl labeling proteomic approaches was performed using PANTHER and DAVID tools. Molecular function analysis revealed that the majority of the differentially expressed proteins demonstrated catalytic (42.93%), binding (26.18%) and structural molecule activities (10.99%) (Figure 3A). The biological processes altered by tachyplesin I treatment were most involved in metabolic processes (30.13%), cellular processes (19.88%), developmental processes (8.43%), localization (8.43%) and biological regulation (8.13%) (Figure 3B). Kyoto Encyclopedia of Genes and Genomes (KEGG) pathways including lysosome pathway (15 proteins), glycosaminoglycan degradation pathway (6 proteins), antigen processing and presentation pathway (8 proteins), DNA replication pathway (5 proteins), type I diabetes mellitus pathway (4 proteins) and glycolysis/gluconeogenesis pathway (4 proteins) are the top pathways altered in response to tachyplesin I treatment (Table 3).

### 2.4. Tachyplesin I Influences Metabolic Process and Alters the Expressions of Cytoskeleton Proteins

In our study, altered proteins involved in metabolic process occupied major share. Of which, glycolytic/gluconeogenesis enzymes including alpha-enolase (ENO1), gamma-enolase (ENO2), triosephosphate isomerase (TPI1) and phosphoglycerate kinase 1 (PGK1) were found to be down-regulated in response to tachyplesin I treatment. In addition, tachyplesin I treatment on U251 gliomaspheres changed the expression of cytoskeleton proteins. Eighteen out of 192 altered proteins induced by tachyplesin I were classified into cytoskeleton protein class in PANTHER classification system. Several cytoskeleton proteins such as spectrin beta chain, non-erythrocytic 2 (SPTBN2), keratin, type II cytoskeletal 1 (KRT1), keratin, type II cytoskeletal 2 epidermal (KRT2), keratin, type I cytoskeletal 9 (KRT9), keratin, type I cytoskeletal 10 (KRT10), vimentin (VIM), ezrin (EZR), interferon-induced GTP-binding protein Mx1 (MX1), interferon-induced GTP-binding protein Mx2 (MX2), cysteine and glycine-rich protein 1 (CSRP1), elongation factor 1-gamma (EEF1G) and tubulin beta-2B chain (TUBB2B) were down-regulated (Table 2) while neurofilament light polypeptide (NEFL), nestin (NES), kinesin-like protein KIF11 (KIF11), tropomyosin alpha-4 chain (TPM4), dystonin (DST) and LIM and cysteine-rich domains protein 1 (LMCD1) were observed with up-regulation (Table 2). To some extent, all these downstream effects of tachyplesin I contribute to its anti-tumor activity.

### 2.5. Tachyplesin I Reduces Expressions of Several Lysosomal Acid Hydrolases

**Figure 4:**
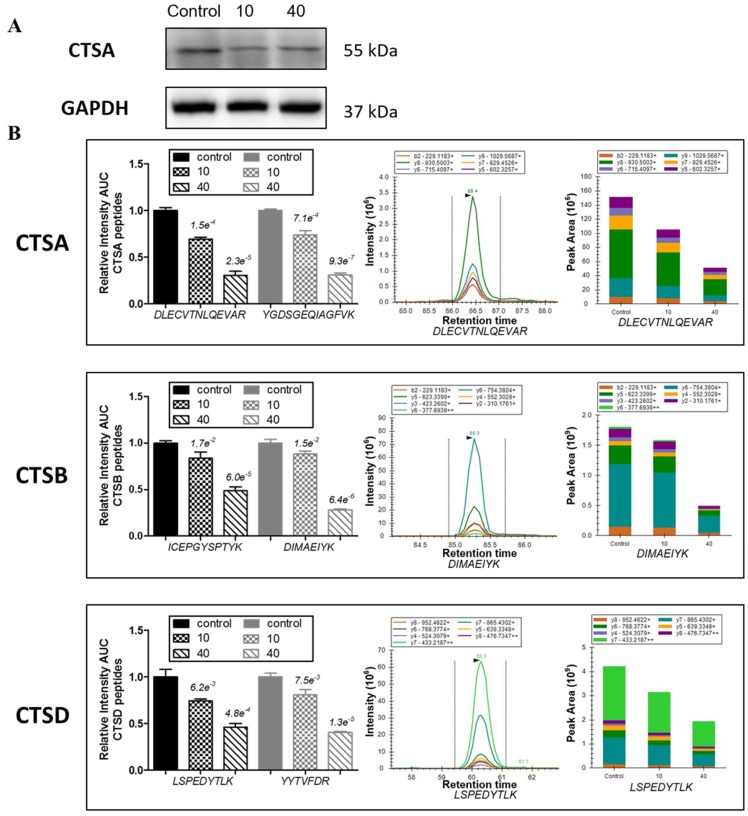
Tachyplesin I reduces expressions of several lysosomal acid hydrolases in U251 gliomaspheres. (**A**) Expression of cathepsin A (CTSA) was validated by Western blot. GAPDH was used as a loading control; (**B**) Expressions of CTSA, cathepsin B (CTSB) and cathepsin D (CTSD) were validated by PRM mass spectrometry. The quantification for two peptides per protein in different dose groups is presented.

As shown in Figure 4A, consistent with the results of proteomic analysis, protein level of lysosomal protective protein (CTSA) was verified to be down-regulated by tachyplesin I using Western blot. Further, other family members of cathepsins, including cathepsin B (CTSB) and cathepsin D (CTSD), as well as cathepsin A (CTSA) were analyzed by PRM mass spectrometry with three technical replicates. For each protein, two unique peptides were selected and monitored for quantification. The skyline software was used to extract the peak areas (area under the curve, AUC) of six to seven strongest transition ions for each peptide (Supplementary Materials Table S5). The normalized sum AUC of all the transitions for each peptide are showed in Figure 4B, which demonstrates that two unique peptides derived from the same protein have a consistent trend, and variations among different technical replicates are small. The results of PRM analysis showed that tachyplesin I down-regulated the levels of CTSA, CTSB and CTSD, which are consistent with dimethyl labeling results.

### 2.6. Protein-Protein Interaction Network of Differentially Expressed Proteins

**Figure 5:**
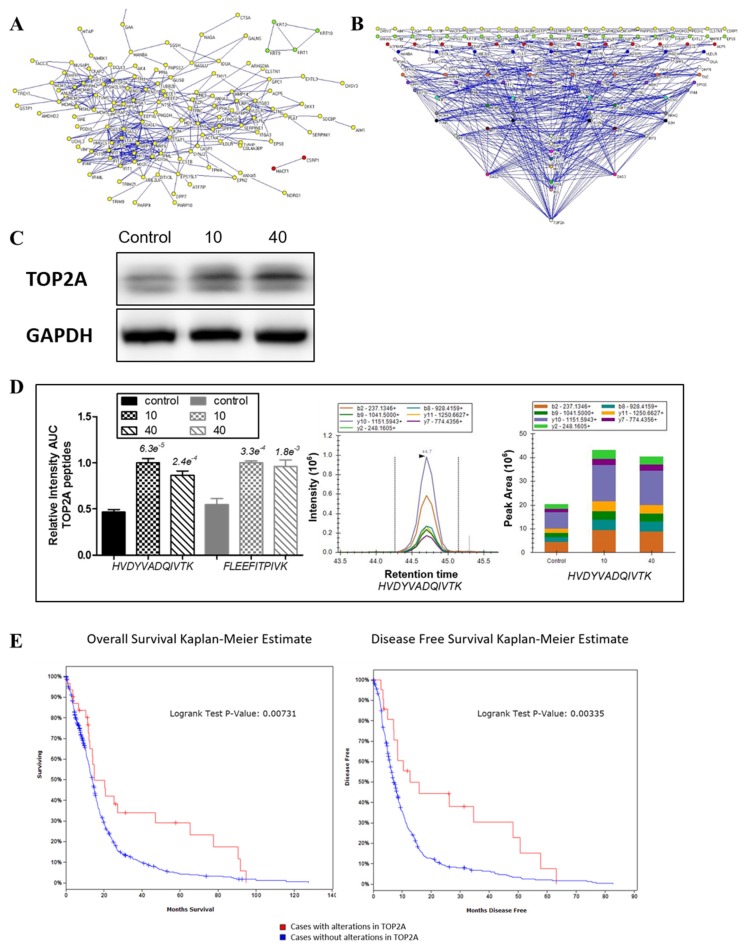
Role of DNA topoisomerase 2-alpha (TOP2A) in protein–protein interaction (PPI) map of tachyplesin I. (**A**) The constructed minimum PPI network of tachyplesin I containing 192 differentially expressed proteins found in 2D-DIGE and dimethyl labeling-based LC-MS analysis; (**B**) Degree distribution map of the proteins in PPI network of tachyplesin I. The proteins are shown as round dots and different colors were only related to degree in the network. TOP2A exhibited to have the biggest degree among all differentially expressed proteins; (**C**) Expression of TOP2A was validated by Western blot. GAPDH was used as a loading control; (**D**) Expression of TOP2A was validated by PRM mass spectrometry. The quantification for two peptides per protein in different dose groups is presented; (**E**) The overall survival (left panel) and the disease-free survival (right panel) of glioma cases with or without alterations in TOP2A. The red curves in the Kaplan–Meier plots includes cases with alterations in TOP2A, the blue curves includes cases without alterations in TOP2A.

Protein–protein interaction (PPI) network was established based on the total 192 differentially expressed proteins related to tachyplesin I treatment, including 26 proteins found in 2D-DIGE analysis and 166 proteins found in dimethyl labeling-based LC-MS analysis. Among them, 180 proteins could connect into a network through direct interaction or an intermediate partner at the PPI level (Figure 5A). Interestingly, DNA topoisomerase 2-alpha (TOP2A) seemed to be the crucial protein in the effects of tachyplesin I as it has the most numerous connections and forms the most complex link with other proteins in the signal network (Figure 5B).

### 2.7. Confirmation of the Involvement of TOP2A in the Effects of Tachyplesin I and Correlation with Clinical Prognosis in TCGA Database

Western blot result (Figure 5C) showed that the expression level of TOP2A was dose-dependent increased after treatment of tachyplesin I, which was consistent with the result of dimethyl labeling based LC-MS/MS analysis (Table 2). At the same time, the expression level of TOP2A was also checked by PRM analysis. As shown in Figure 5D, after tachyplesin I treatment, the expression level of TOP2A was up-regulated, which verified the data obtained from dimethyl labeling based quantification. Then, we used cBioPortal tool to analyze the relationship between the mRNA transcript level of TOP2A and clinical prognosis of GBM patients based on TCGA database to examine the effects of tachyplesin I by targeting on TOP2A. As shown in Figure 5E, patients with alterations in TOP2A at mRNA transcript level have a better prognosis compared with those without alterations in TOP2A. The analysis showed a significantly better overall and disease-free survival of patients with over-expression of TOP2A.

## 3. Discussion

More and more studies have shown that certain cationic antimicrobial peptides (AMPs), which are toxic to bacteria but not to normal mammalian cells, exhibit a broad spectrum of cytotoxic activity against cancer cells [20]. Tachyplesin I, which is isolated from hemocytes of the horseshoe crab, has been identified as a member of AMPs and exhibits cytotoxic activity against cancer cells. However, it is uncertain why only some types of AMPs get kill cancer cells, while others not. Besides, whether the molecular mechanisms underlying the antitumor and antimicrobial activities are the same or not remains unclear. Through this study we aim to identify the protein targets of tachyplesin I in U251 gliomaspheres by carrying out a large-scale proteome analysis, which can help us to better understand the molecular mechanisms underlying AMPs as potential anti-glioma drugs.

In this study, gel-based 2D-DIGE and stable isotope dimethyl labeling based LC-MS/MS analysis were combined to reveal the alteration in proteome of U251 gliomaspheres treated with tachyplesin I. A total of 192 differentially expressed proteins were identified, most of which are involved in the cellular process of metabolism, especially glycolysis process, and many proteins are localized as cytoskeleton proteins and lysosomal acid hydrolases. Especially, the expression level of some proteins of interest was validated by PRM, a high-resolution method first published in 2012 and had several potential advantages over traditional approach [21]. For example, PRM spectra are highly specific as a result of all the product ions of a peptide are recorded to confirm peptide identity, while traditional MRM analysis can only monitor one transition of a precursor peptide at a time. Moreover, high-resolution of the orbitrap mass analyzer can separate co-eluted background ions, thus increasing selectivity [22].

One of the hallmarks of tumor cells is the preference of glycolysis over oxidative phosphorylation as the main source of energy. Although glycolysis yield less ATP compared to oxidative phosphorylation with the same amount of beginning materials, tumor cells overcome this disadvantage by increasing the up-take of glucose, thus facilitates a higher rate of glycolysis [23]. Studies have showed that glycolysis plays a role in the invasion activity of glioma cells and is becoming a potential drug target [24]. In this study, glycolytic/gluconeogenesis enzymes including alpha-enolase (ENO1), gamma-enolase (ENO2), triosephosphate isomerase (TPI1) and phosphoglycerate kinase 1 (PGK1) were down-regulated in response to tachyplesin I treatment, indicating that tachyplesin I may disrupt the normal energy metabolism process in gliomaspheres through reduced glycolysis, thus contributing to its anti-tumor effect.

Uncontrolled and invasive proliferation is one feature of grade IV glioma, and in order to block and restrain mitotic division, cytoskeleton has been a time-honored target in cancer chemotherapy [25]. In this study, tachyplesin I treatment on U251 gliomaspheres altered the expression of 18 cytoskeleton proteins as classified by PANTHER classification system. Among them, vimentin and ezrin, which are known to be involved in the regulation of metastasis, were down-regulated under the treatment of tachyplesin I, suggesting that cytoskeleton are influenced by tachyplesin I, thus contributes to its anti-tumor activity.

Out of 192 altered proteins, 15 are lysosomal acid hydrolases, including proteases, glycosidases, sulfatases, lipases and so on. In addition, DAVID pathway classification system revealed lysosome as the most significantly altered pathway. More and more experimental evidences suggest that tumor invasion and metastasis are associated with alterations in lysosomes and increased expression of the lysosomal proteases termed cathepsins [26]. In this study, cathepsins consist of cathepsin A, B and D were down-regulated in response to tachyplesin I treatment. Cathepsin A, also called lysosomal protective protein, is a serine carboxypeptidase implicated in autophagy. It induces tumor cell dissemination and a significant increase in cathepsin A activity in lysates of metastatic lesions of malignant tumor was observed compared to primary focus lysates [27]. Cathepsin B is a lysosomal cysteine protease of the papain family of enzymes that function as an endopeptidase and an exopeptidase [28]. Cathepsin D, an aspartic protease resides in membrane of lysosomes, is involved in autophagy and apoptosis pathways [29]. Interestingly, it has been shown that cathepsin B and D play an important role in human glioma progression and invasion [30]. The expression and enzyme activity of cathepsin B and D gradually increased in high-grade glioblastoma. Inhibition of cathepsin B or D activity attenuates extracellular matrix degradation thus reduces migration of glioma cells [31]. Our data showed that the levels of these cathepsins were significantly decreased in tachyplesin I-treated gliomaspheres compared with untreated cells. All those evidences indicate the potential of tachyplesin I as a therapeutic agent for glioma by targeting the lysosomal activity.

In further PPI analysis of differentially expressed proteins, DNA topoisomerase 2-alpha (TOP2A) was shown to be the possible critical target protein of tachyplesin I. TOP2A is a nuclear enzyme for regulation of DNA topology and replication. TOP2A was discovered to be the target of many anti-tumor drugs which had already been widely used in clinic. Previous reports have shown that DNA damage and fragmentation induced by covalent binding of TOP2A to DNA, and forced expression of TOP2A in cells triggered the apoptotic cell death [32,33]. In addition, the TOP2A level has a close relationship with the activity of these anti-tumor drugs and a high level of TOP2A is the foundation of drug susceptibility. Meanwhile, decreased level, altered phosphorylation or mutation of TOP2A could induce the loss of anti-tumor drug target and develop multiple drug resistance (MDR), which has been confirmed in atypical MDR studies with many cell lines [34,35]. Our results showed that there was an increased TOP2A level in U251 gliomaspheres treated with tachyplesin I and it suggest the possible synergistic effect with TOP2A-targeting drugs, combination of which may be more effective on targeted goals and improve chemotherapy effect.

## 4. Material and Methods

### 4.1. Cell Culture and Treatment with Tachyplesin I

U251 human glioma cells were obtained from the Chinese Academy of Sciences Cell Band (Shanghai, China) and cultured in RPMI-1640 medium containing 10% fetal bovine serum (FBS) and 100 units/mL penicillin/streptomycin at 37 °C in a humidified atmosphere with 5% CO_2_. The U251 cells in the logarithmic growth phase were thoroughly dissociated to prepare single-cell suspensions. Cell suspensions were washed twice in PBS and resuspended in Neurobasal-A medium with 1× B27 plus 50 ng/mL basic fibroblast growth factor (bFGF) and 50 ng/mL epidermal growth factor (EGF). After 7 days culture, clones of different morphological types were collected. The obtained cells which exhibited certain glioma stem cell phenotypes [11] were cultured as gliomaspheres and passaged every 7 days, based on sphere size.

Tachyplesin I was synthesized by Hanyu Bioengineering Company (Shenzhen, China) with a purity of >95%. Concentrations of tachyplesin I for cell exposure were determined by cell viability assay as described previously [11]. The second generation gliomaspheres were treated with 0, 10, 40 and 80 μg/mL of tachyplesin I for 24 h, and then cells were centrifuged and collected.

### 4.2. CyDye Minimal Labeling of Protein Samples and 2D-DIGE Electrophoresis

Proteins were extracted from gliomaspheres treated with 0, 10, 40 and 80 μg/mL of tachyplesin I using the lysis buffer containing 7 M urea, 2 M thiourea, 4% (*w*/*v*) CHAPS and 30 mM Tris-HCl. The concentrations of proteins were determined with the 2-D Quant kit (GE Healthcare, Piscataway, NJ, USA) according to the manufacturer’s instructions. Then an equal amount (25 μg) of each protein sample was minimally labeled with Cy3 or Cy5 fluorescent dyes (GE Healthcare) according to the manufacturer’s recommended protocols and the internal standard, resulting from pooling equal aliquots of all experimental samples, was labeled with Cy2.

Six differently pooled samples (Table 4), which comprised equal amounts of Cy3- and Cy5-labeled protein samples and Cy2-labeled internal standard, were then separated by first dimension of isoelectric focusing using 24 cm IPG strips (pH 3–11, nonlinear gradient, GE Healthcare), followed by second dimension separation into 12.5% SDS-PAGE gels. Gels were then scanned with different channels for Cy2-, Cy3-, and Cy5-labeled proteins, using a Typhoon Trio Variable Mode Imager (GE Healthcare). The resulting 18 maps were imported into DeCyder 2D v6.5 (GE Healthcare) for statistical analysis. Each gel was separately processed by the Differential In-gel Analysis (DIA) module for spot detection, background subtraction and in-gel normalization before processed by the Biological Variation Analysis (BVA) module for spot matching and intercomparison across the six gels. Student’s *t*-test was used to analyze the significance of protein spots between two groups, and one way ANOVA was subsequently used to assess the biological significance among all the experimental groups. Statistically significant spots (*p* < 0.05) with an average ratio ≥1.5 or ≤−1.5 were chosen for protein identification.

### 4.3. In-Gel Digestion and Protein Identification by MALDI-TOF/TOF

For identification of spots of interest, a gel was prepared by separating 1 mg of unlabeled proteins pooled from all the samples. The gel was stained by Coomassie Brilliant Blue G-250 and destained by water to reveal the protein spots. After matching to the analytical DIGE gel, each spot of interest was manually excised from the gel and put into a 1.5 mL tube, followed by thorough decoloration with 50% acetonitrile in 25 mM ammonium bicarbonate and dehydration in 100% acetonitrile. Then each gel piece was digested overnight at 37 °C by trypsin in 25 mM ammonium bicarbonate buffer. Peptides were extracted from each gel piece, desalted, and identified by an UltrafleXtreme MALDI-TOF/TOF mass spectrometer (Bruker Daltonics, Billerica, MA, USA) according to previously described [20].

### 4.4. Dimethyl Labeling of Protein Samples

Cells were lysed with a lysis buffer containing 4% SDS, 100 mM Tris, pH 8.0 and 1× protease inhibitor cocktail (Roche, Indianapolis, IN, USA). Protein concentrations were determined using a Pierce BCA Protein Assay Kit (Thermo Fisher Scientific, Waltham, MS, USA). One milligram protein from each sample was reduced with 5 mM dithiothreitol, alkylated with 15 mM iodoacetamide, and precipitated by methanol and chloroform [36]. The resulting pellets were resuspended in lysis buffer containing 8 M urea, 0.1 M Tris-HCl, pH 8.5 and the concentration of urea was diluted to below 2 M before overnight digestion with trypsin (Promega, Madison, WI, USA).

Dimethyl labeling was performed on-column according to Nature Protocols by Boersema P.J. et al. [37] with minor modifications. Briefly, acidified peptide samples were loaded into SepPak columns (Waters, Milford, MA, USA) after the columns were activated by methanol, 80% acetonitrile in 0.1% trifluoroacetic acid (TFA), and conditioned by 0.1% TFA. After desalting, the samples were labeled separately by passing the columns with CH_2_O and NaBH_3_CN (light), CD_2_O and NaBH_3_CN (medium) and CD_2_O and NaBD_3_CN (heavy) (Sigma-Aldrich, St. Louis, MO, USA) for 20 min at room temperature. Labeling scheme was shown in Table 5. Then the differentially labeled samples were eluted from the columns, mixed and dried by Speedvac (Labconco, Kansas, MO, USA).

### 4.5. High pH Fractionation of Peptides and LC-MS/MS Analysis by Obitrap

The dimethyl-labeled sample was resuspended in 1% formic acid (FA), loaded into SepPak column, and fractionated into five fractions by eluting the peptides with 3%, 6%, 9%, 15% and 80% (vol/vol) acetonitrile in 5 mM ammonium formate (pH 10.0), sequentially. After lyophilization in Speedvac, samples were resuspended in 0.1% FA and analyzed by a Q-Exactive orbitrap mass spectrometer (Thermo Fisher Scientific) coupled to an Easy-nLC 1000 (Thermo Fisher Scientific) ultrahigh pressure liquid chromatography (UHPLC). The LC separation system consisted of a trap column (100 μm i.d. × 4 cm) and an analytical column (75 μm i.d. × 20 cm) both packed with 3 μm/120 Å C18 resins (Dr. Maisch HPLC GmbH, Ammerbuch, Germany). The eluting buffers were 0.1% FA in H_2_O (buffer A) and 0.1% FA in 99.9% ACN (buffer B). The peptides were first loaded onto the trap column and then separated by the analytical column with 50 min gradient from 7% to 22% buffer B followed by 10 min gradient from 22% to 35% buffer B at a flow rate of 300 nL/min. MS data was acquired in data dependent acquisition (DDA) mode. Survey full scan MS spectra (*m*/*z* 350–1550) were acquired in the Orbitrap with resolution of 70,000, target automatic gain control (AGC) value of 3 × 10^6^, and maximum injection time of 100 ms. Dynamic exclusion for scanned presursors was employed for 60 s. After each MS scan, the 10 most intense precursor ions (*z* ≥ 2) were sequentially isolated and fragmented by higher-energy collisional dissociation (HCD) using normalized energy 27% with an AGC target of 1 × 10^5^ and a maxima injection time of 50 ms at 17,500 resolution.

Raw data were searched through UniProt Homo sapiens protein database containing 70,076 sequence entries via Sequest HT algorithm with the following parameters: two missed cleavage sites by trypsin, 10 ppm mass tolerance for precursors, 0.02 Da mass tolerance for fragments, and carbamidomethylation (+57.021 Da) of cysteineas static modifications. Moreover, the following dynamic modifications were also set: oxidation of methionine (+15.995 Da), deamidation of asparagine or glutarnine (+0.984 Da), and dimethylation for light-labeled (+28.031 Da) or medium-labeled (+32.056 Da) or heavy-labeled (+36.076 Da) lysine, and *N*-terminus. All the identified peptides were filtered by FDR <0.01 as reliable identification. Protein Discoverer was used for relative quantification. Differentially expressed proteins were considered for ratios ≤0.5 (down-regulated) and ≥2 (up-regulated).

### 4.6. Bioinformatic Analysis

The function reports of the candidate proteins whose expression was altered in U251 gliomaspheres due to the effect of tachyplesin I treatment were obtained from the UniProt database (http://www.uniprot.org/) and the protein list of UniProt IDs was input into the PANTHER classification system (http://pantherdb.org/) for GO analysis according to their molecular functions and biological processes. The relevant signaling pathways highly associated with the effect of tachyplesin I treatment on U251 gliomaspheres were identified using DAVID analysis (https://david.ncifcrf.gov/). The protein–protein interaction network of all the differentially expressed proteins was established using String (http://string-db.org/), and then the data was exported as .net file and imported into pajek software for degree based partition of the proteins in the network. The correlation of the possible key proteins involved in the effects of tachyplesin I in our proteomic analysis with its mRNA transcript level and clinical prognosis in GBM patients based on per TCGA data was analyzed by cBioPortal tools (http://www.cbioportal.org/) [38].

### 4.7. Parallel Reaction Monitoring (PRM) Mass Spectrometry

We applied PRM to validate the major protein changes observed in the dimethyl labeling analyses. Proteins were extracted from another batch of differently treated U251 gliomaspheres (biological replicate) and digested to peptides. These unlabeled peptides were fractionated and identified as described above with only difference in database searching (no dimethylation as dynamic modifications). For PRM analysis, 2 μg of non-fractionated peptides from each group were separated using the same LC system. Linear gradient ranging from 4% to 35% buffer B over 60 min was used. For each target protein, two unique precursor peptide ions were monitored in the inclusion list. The settings for MS full scan were the same as in the DDA mode with only different in *m*/*z* scan range (300–900). The following MS/MS PRM scan parameters were set: orbitrap resolution of 35,000, AGC target value of 5 × 10^5^, auto maximum IT, isolation window of 2 *m*/*z*, HCD collision energy of 27, and starting mass of *m*/*z* 110. The PRM raw files were analysed using Skyline [39] to extract the peak areas of six to seven most intense transitions for each peptide. Then the data was imported to GraphPad for statistical analysis. Differences between two groups were analyzed by the Student’s *t*-test and statistical significance was considered when *p* < 0.05.

### 4.8. Western Blot Assay

Total proteins were extracted from different groups of U251 gliomaspheres with the same treatment as described in the DIGE analysis, and protein concentrations were quantified by BCA kit. Western blot procedures were carried out as we previously described [40], with minor modifications. Namely, after boiling for 5 min with loading buffer, the same amount of proteins from each groups were separated by SDS-PAGE and transferred onto PVDF membranes. The membranes were incubated with mouse monoclonal anti-ECE-1 antibody (sc-376017, Santa Cruz, CA, USA), mouse monoclonal anti-alpha-enolase (sc-101513, Santa Cruz, CA, USA), mouse monoclonal anti-cathepsin A (sc-73766, Santa Cruz, CA, USA) and mouse monoclonal anti-GAPDH (sc-32233, Santa Cruz, CA, USA) at 1:500 dilution. The immunoblots were developed by incubation with goat anti-mouse IgG-HRP (sc-2005, Santa Cruz, CA, USA) as the secondary antibody followed by ECL detection (GE Healthcare).

## 5. Conclusions

In our study, we combined a gel-based 2D-DIGE approach and a dimethyl labeling LC-MS-based shotgun proteomic strategy to identify the proteome expression alterations in U251 gliomaspheres treated with different doses of tachyplesin I. Our results demonstrate complementary advantages of these two techniques. We show that tachyplesin I alters the cellular metabolism, especially glycolysis process and changes the expression of several cytoskeleton proteins and lysosomal acid hydrolases. Moreover, the important role of DNA topoisomerase 2-alpha (TOP2A) in the signal cascades of tachyplesin I was suggested. Further, parallel reaction monitoring (PRM) mass spectrometry confirmed that the major protein of lysosomal acid hydrolases including cathepsin A, cathepsin B and cathepsin D were down-regulated and the possible target-related protein TOP2A was up-regulated by tachyplesin I treatment. In conclusion, we propose that tachyplesin I may down-regulate cathepsins in lysome and up-regulate TOP2A to inhibit migration and promote apoptosis in glioma, thus contributing to its anti-tumor activity. Further work including functional analyses is needed to elucidate the mode of action of tachyplesin I in tumor cells. As far as we know, there is no previous report that reveals the effect of tachyplesin I on proteome of gliomaspheres and our findings imply that tachyplesin I could serve as a promising candidate in the combined therapy against glioma.

## Figures and Tables

**Table 1:** Regulated proteins of tachyplesin I treated U251 gliomaspheres in the 2D-DIGE study.

Up-Regulated Proteins of Tachyplesin I Treated U251 Gliomaspheres in the 2D-DIGE Study									
No. ^a^	Gene Name	Uniprot ID	Protein Name	Mascot Score	Peptides	Protein MW	pI Value	Ratio/*p* Value ^b^	Ratio/*p* Value ^b^
								10 vs. 0 ^c^	40 vs. 0 ^c^
Regulation of cell cycle or apoptosis ^d^									
2	PHGDH	O43175	d-3-phosphoglycerate dehydrogenase	104	3	57,356	6.3	1.54/0.003	1.69/0.017
5	MSH2	P43246	DNA mismatch repair protein Msh2	93	2	104,743	5.8	1.21/0.009	1.77/0.035
19	SESN3	P58005	Sestrin-3	201	4	57,291	6.3	1.52/0.036	2.01/0.027
31	CKAP2	Q8WWK9	Cytoskeleton-associated protein 2	76	7	76,987	9.4	ND ^e^	1.52/0.039
35	ECE1	P42892	Endothelin-converting enzyme 1	46	1	87,164	5.9	1.58/0.038	3.31/0.026
Cytoskeletal protein ^d^									
3	VIM	P08670	Vimentin	407	12	53,676	4.9	ND	1.64/0.022
4	EEF1G	P26641	Elongation factor 1-gamma	47	2	50,429	6.3	ND	1.51/0.005
9	EZR	P15311	Ezrin	168	3	69,484	5.9	1.03/0.024	1.63/0.046
10	VIM	P08670	Vimentin	524	15	53,676	4.9	ND	1.58/0.041
Protein biosynthesis ^d^									
6	EEF2	P13639	Elongation factor 2	60	1	96,246	6.4	ND	1.55/0.044
21	PPIA	P62937	Peptidyl-prolyl *cis*-*trans* isomerase A	195	16	18,229	9	1.26/0.017	1.52/0.028
Transport ^d^									
23	SLC25A3	F8VVM2	Phosphate carrier protein, mitochondrial	90	5	36,161	9.3	ND	1.72/0.034
25	SLC25A3	F8VVM2	Phosphate carrier protein, mitochondrial	183	9	36,161	9.3	ND	1.63/0.016
**Down-Regulated Proteins of Tachyplesin I Treated U251 Gliomaspheres in the 2D-DIGE Study**									
Calcium or iron ion binding protein ^d^									
7	EPS15	P42566	Epidermal growth factor receptor substrate 15	109	5	98,656	5.1	ND ^e^	−1.88/0.004
13	P4HA1	P13674	Prolyl 4-hydroxylase subunit alpha-1	86	5	61,296	5.6	−1.51/0.007	−2.37/0.045
Regulation of cell apoptosis or proliferation ^d^									
12	ANXA5	P08758	Annexin A5	273	13	35,971	4.8	ND	−1.74/0.033
20	GSTP1	P09211	Glutathione *S*-transferase P	339	21	23,569	5.3	ND	−1.66/0.001
33	COL4A3BP	Q9Y5P4	Collagen type IV alpha-3-binding protein	250	7	70,835	5.5	ND	−1.64/0.033
34	ARHGDIA	P52565	Rho GDP-dissociation inhibitor 1	236	16	23,250	4.9	−1.23/0.037	−2.64/0.047
Response to stimulus ^d^									
14	GNAQ	P50148	Guanine nucleotide-binding protein G(q) subunit alpha	193	9	42,142	5.7	ND	−1.61/0.037
16	GNAQ	P50148	Guanine nucleotide-binding protein G(q) subunit alpha	294	12	42,142	5.7	ND	−1.59/0.047
28	GNAQ	P50148	Guanine nucleotide-binding protein G(q) subunit alpha	182	7	42,142	5.7	−1.33/0.028	−1.53/0.036
Glycolysis/Gluconeogenesis ^d^									
15	ENO1	P06733	Alpha-enolase	40	3	47,481	7.7	−1.04/0.005	−1.92/0.054
17	PGK1	P00558	Phosphoglycerate kinase 1	209	7	44,985	9.2	−1.68/0.025	−2.89/0.051
30	TPI1	P60174	Triosephosphate isomerase	375	15	31,057	5.6	−1.17/0.048	−1.88/0.028
Ribosomal protein ^d^									
18	RPSA	P08865	40S ribosomal protein SA	171	9	32,947	4.6	−1.49/0.036	−1.89/0.027

^a^ No.—The numbers correspond to the spot numbers indicated in Figure 1B; ^b^ Average ratios of spot abundance of tachyplesin I-treated samples relative to the control, represent data from three separate experiments and student’s *t* test *p* values are given as a measure of confidence for the ratio of each spot measured; ^c^ 0: control group; 10: 10 μg/mL dose group; 40: 40 μg/mL dose group; ^d^ Functional categories according to Gene ontology and panther biological process annotations; ^e^ ND, not detected or *p* value > 0.5.

**Table 2:** List of proteins with altered expression in U251 gliomaspheres after treatment of tachyplesin I using dimethyl labeling quantitative proteomic analysis.

The 55 Up-Regulated Proteins Expressed More Than 2 Folds (<1% FDR)									
Gene Name	Uniprot ID	Protein Name	Coverage (%) ^a^	Unique Peptides ^a^	10 vs. 0 Ratio ^b^		40 vs. 0 Ratio ^b^		Protein Class ^c^
					Forward	Reverse	Forward	Reverse	
SPP1	P10451	Osteopontin	33.12	5	4.961	2.943	12.876	9.484	cytokine
ITGB3	P05106	Integrin beta-3	5.20	3	4.754	5.953	13.279	28.714	receptor, extracellular matrix glycoprotein
EPS8	Q12929	Epidermal growth factor receptor kinase substrate 8	4.01	3	4.403	2.549	2.220	2.305	transmembrane receptor regulatory/adaptor protein
MCM5	B1AHB1	DNA helicase	5.50	3	3.304	2.022	3.801	2.194	DNA helicase
DKK1	O94907	Dickkopf-related protein 1	11.28	4	3.267	2.278	3.885	2.732	developmental protein, growth factor activity
MCM4	P33991	DNA replication licensing factor MCM4	7.76	4	3.248	2.119	5.189	3.723	DNA binding protein
NUSAP1	Q9BXS6	Nucleolar and spindle-associated protein 1	18.14	5	2.661	2.293	3.121	3.405	microtubule-associated protein
DHFR	P00374	Dihydrofolate reductase	24.06	4	2.616	2.168	2.398	2.472	reductase
TOP2A	P11388	DNA topoisomerase 2-alpha	12.93	12	2.490	2.916	3.259	2.582	DNA topoisomerase, enzyme modulator
MKI67	A0A087WV66	Antigen KI-67	12.66	24	2.396	1.870	2.688	2.047	regulation of cell proliferation
TFRC	P02786	Transferrin receptor protein 1	35.79	23	2.323	2.398	2.906	2.879	receptor
AIM1	Q9Y4K1	Absent in melanoma 1 protein	21.53	25	2.298	2.329	4.036	5.403	carbohydrate binding protein
ECE1	P42892	Endothelin-converting enzyme 1	15.19	8	2.260	2.671	3.557	4.070	metalloprotease
SYNJ2	O15056	Synaptojanin-2	8.76	11	2.239	2.501	5.349	4.740	phosphatase
KIF11	P52732	Kinesin-like protein KIF11	2.37	2	2.199	2.274	2.469	2.182	microtubule binding motor protein
DST	Q03001	Dystonin	23.49	21	2.113	1.596	2.614	2.051	non-motor actin binding protein
UPP1	Q16831	Uridine phosphorylase 1	46.45	11	2.078	2.712	2.209	3.577	phosphorylase
IGFBP5	P24593	Insulin-like growth factor-binding protein 5	20.59	5	2.070	2.060	4.816	4.967	cell communication
RRM2	P31350	Ribonucleoside-diphosphate reductase subunit M2	34.45	11	2.030	1.981	2.286	2.423	reductase
CD70	P32970	CD70 antigen	35.23	6	1.995	2.167	3.725	3.808	cell communication
MDK	E9PPJ5	Midkine (Fragment)	27.48	2	1.935	2.538	3.569	4.336	cytokine
HMGCS1	Q01581	Hydroxymethylglutaryl-CoA synthase, cytoplasmic	42.88	17	1.929	1.939	3.322	4.339	transferase, lyase
DCLK1	Q5VZY9	Serine/threonine-protein kinase DCLK1	10.74	3	1.899	3.222	4.721	8.819	non-receptor serine/threonine protein kinase
MCM7	P33993	DNA replication licensing factor MCM7	13.21	7	1.879	1.963	2.451	2.470	DNA helicase
PODXL	O00592	Podocalyxin	2.33	1	1.870	2.234	2.474	2.856	regulation of adhesion and cell morphology
MCM2	H0Y8E6	DNA replication licensing factor MCM2 (Fragment)	8.25	6	1.865	1.513	2.167	2.664	DNA helicase
LPL	P06858	Lipoprotein lipase	34.11	12	1.848	2.078	4.136	4.351	storage protein
VSNL1	P62760	Visinin-like protein 1	24.08	4	1.805	2.260	3.231	2.926	cell communication
MCM6	Q14566	DNA replication licensing factor MCM6	9.01	4	1.765	2.208	2.606	3.031	DNA helicase
GPC1	P35052	Glypican-1	33.69	14	1.745	1.665	2.861	3.347	cell division and growth regulation
TACC3	Q9Y6A5	Transforming acidic coiled-coil-containing protein 3	3.22	2	1.735	2.068	2.563	2.322	cytoskeleton
TNC	P24821	Tenascin	40.16	5	1.730	1.803	2.327	2.112	signaling molecule
PLAT	P00750	Tissue-type plasminogen activator	24.73	12	1.729	3.475	8.267	13.172	receptor, calmodulin
GATM	P50440	Glycine amidinotransferase, mitochondrial	28.61	9	1.704	1.793	2.591	2.740	catalyze creatine biosynthesis
SERPINE1	P05121	Plasminogen activator inhibitor 1	21.14	7	1.695	1.448	4.539	3.853	serine protease inhibitor
LMCD1	Q9NZU5	LIM and cysteine-rich domains protein 1	38.63	10	1.681	1.686	2.388	2.243	structural protein
TYMS	P04818	Thymidylate synthase	19.17	4	1.675	2.752	2.151	3.228	methyltransferase
ITGA3	P26006	Integrin alpha-3	21.41	19	1.671	1.729	2.847	2.944	receptor, integrin
ANLN	Q9NQW6	Actin-binding protein anillin	3.91	3	1.666	2.235	2.308	3.143	actin binding protein
ANXA2	P07355	Annexin A2	81.42	34	1.617	1.610	2.023	2.100	fatty acid metabolic process
MACF1	H3BPE1	Microtubule-actin cross-linking factor 1, isoforms 1/2/3/5	29.64	153	1.610	1.601	2.018	2.000	non-motor actin binding protein
TPM4	P67936	Tropomyosin alpha-4 chain	48.79	8	1.534	2.126	2.878	2.893	actin binding motor protein
ACTN4	K7EJH8	Alpha-actinin-4 (Fragment)	68.68	1	1.526	2.411	2.251	3.153	non-motor actin binding protein
TRIM9	Q9C026	E3 ubiquitin-protein ligase TRIM9	4.23	3	1.508	1.143	2.077	2.109	ubiquitin-protein ligase
LDLR	P01130	Low-density lipoprotein receptor	6.63	5	1.498	1.202	2.266	2.096	receptor, extracellular matrix glycoprotein
SDCBP	O00560	Syntenin-1	28.52	4	1.495	1.490	2.581	3.361	membrane trafficking regulatory protein
TF	P02787	Serotransferrin	45.13	27	1.422	1.526	2.224	2.216	transfer/carrier protein
TENM2	H7BYZ1	Teneurin-2	13.86	24	1.422	1.735	2.046	2.501	receptor, membrane-bound signaling molecule
NES	P48681	Nestin	58.61	78	1.396	1.363	2.140	2.167	structural protein
THY1	E9PIM6	Thy-1 membrane glycoprotein (Fragment)	25.66	3	1.360	1.468	2.432	2.131	membrane glycoprotein
NEFL	P07196	Neurofilament light polypeptide	47.88	28	1.213	1.182	2.121	2.010	structural protein
CLSTN1	Q5SR54	Calsyntenin-1 (Fragment)	4.35	3	1.181	1.201	2.338	2.552	cell adhesion molecule, calcium-binding protein
ECI2	A0A0C4DGA2	Enoyl-CoA delta isomerase 2, mitochondrial	40.38	11	1.148	1.262	2.239	2.894	transfer/carrier protein, enzyme modulator
PTPRE	P23469	Receptor-type tyrosine-protein phosphatase epsilon	11.29	5	0.939	2.069	3.282	3.385	receptor, protein phosphatase
LRRC16A	Q5VZK9	Leucine-rich repeat-containing protein 16A	1.90	2	ND	1.408	2.281	3.321	transcription cofactor
**The 111 Down-Regulated Proteins Expressed Less Than 0.5 Folds (<1% FDR)**									
OASL	Q15646	2′-5′-oligoadenylate synthase-like protein	12.26	4	0.127	0.393	0.215	ND	nucleotidyltransferase, defense/immunity protein
OAS2	P29728	2′-5′-oligoadenylate synthase 2	9.74	9	0.151	0.158	0.115	0.068	nucleotidyltransferase, defense/immunity protein
MX1	P20591	Interferon-induced GTP-binding protein Mx1	56.50	28	0.164	0.177	0.150	0.130	microtubule family cytoskeletal protein
IFI44L	Q53G44	Interferon-induced protein 44-like	39.60	13	0.179	0.206	0.189	0.191	immune response
IFI44	Q8TCB0	Interferon-induced protein 44	33.56	13	0.193	0.231	0.158	0.181	immune response
CASP1	G3V169	Caspase	19.35	4	0.209	0.325	0.235	0.180	regulation of apoptotic process
BTN3A2	E9PRR1	Butyrophilin subfamily 3 member A2 (Fragment)	27.55	2	0.220	0.555	0.261	0.360	ubiquitin-protein ligase
INS	C9JNR5	Insulin (Fragment)	7.61	1	0.228	0.231	0.634	0.769	growth factor
MX2	P20592	Interferon-induced GTP-binding protein Mx2	16.05	5	0.235	0.140	0.129	0.215	microtubule family cytoskeletal protein
PARP10	E9PPE7	Poly [ADP-ribose] polymerase 10	4.71	2	0.260	0.280	0.201	0.508	nucleic acid binding
ISG15	A0A096LNZ9	Ubiquitin-like protein ISG15 (Fragment)	50.35	6	0.273	0.293	0.253	0.264	ribosomal protein
TAP1	Q03518	Antigen peptide transporter 1	29.08	15	0.287	0.385	0.288	0.288	ATP-binding cassette (ABC) transporter
IFIT3	O14879	Interferon-induced protein with tetratricopeptide repeats 3	48.57	18	0.288	0.301	0.261	0.258	RNA binding
IFIT2	P09913	Interferon-induced protein with tetratricopeptide repeats 2	30.08	11	0.294	0.291	0.212	0.250	RNA binding
IFIT1	P09914	Interferon-induced protein with tetratricopeptide repeats 1	45.82	16	0.300	0.321	0.296	0.301	RNA binding
KRT10	P13645	Keratin, type I cytoskeletal 10	30.14	13	0.301	0.314	0.483	0.541	structural protein
DDX58	O95786	Probable ATP-dependent RNA helicase DDX58	41.73	37	0.307	0.307	0.278	0.265	helicase, hydrolase
BLOC1S1	G8JLQ3	Biogenesis of lysosome-related organelles complex 1 subunit 1	50.67	3	0.308	0.461	0.296	0.417	transcription factor
TRIM21	P19474	E3 ubiquitin-protein ligase TRIM21	7.79	3	0.310	0.382	0.259	0.340	ubiquitin-protein ligase
OAS3	Q9Y6K5	2′-5′-oligoadenylate synthase 3	30.08	29	0.316	0.306	0.246	0.237	nucleotidyltransferase, defense/immunity protein
SLC4A4	Q9Y6R1	Electrogenic sodium bicarbonate cotransporter 1	9.64	8	0.323	0.290	0.164	0.214	transporter
TAPBP	O15533	Tapasin	25.00	7	0.324	0.397	0.318	0.333	immunoglobulin receptor superfamily
KRT1	P04264	Keratin, type II cytoskeletal 1	36.49	18	0.325	0.273	0.550	0.447	structural protein
DTX3L	Q8TDB6	E3 ubiquitin-protein ligase DTX3L	25.81	12	0.326	0.426	0.375	0.377	ubiquitin-protein ligase
TAP2	Q03519	Antigen peptide transporter 2	22.16	10	0.350	0.350	0.280	0.270	ATP-binding cassette (ABC) transporter
GBP1	P32455	Interferon-induced guanylate-binding protein 1	28.38	13	0.362	0.346	0.296	0.202	heterotrimeric G-protein
KRT9	P35527	Keratin, type I cytoskeletal 9	35.47	15	0.363	0.409	0.632	0.759	structural protein
AGTRAP	Q6RW13	Type-1 angiotensin II receptor-associated protein	13.84	1	0.383	0.479	0.557	0.604	response to hypoxia
PARP9	Q8IXQ6	Poly [ADP-ribose] polymerase 9	16.28	12	0.387	0.392	0.395	0.359	nucleic acid binding
HLA-B	P30466	HLA class I histocompatibility antigen, B-18 alpha chain	57.73	1	0.396	0.414	0.307	0.344	immunoglobulin receptor superfamily
IRF9	Q00978	Interferon regulatory factor 9	7.38	3	0.405	0.588	0.318	0.377	immune response
C19orf66	Q9NUL5	UPF0515 protein C19orf66	16.15	3	0.405	0.480	0.214	0.395	no function identified yet
NT5E	P21589	5′-nucleotidase	48.08	25	0.408	0.428	0.353	0.361	nucleotide phosphatase
STAT1	P42224	Signal transducer and activator of transcription 1-alpha/beta	53.33	36	0.408	0.422	0.369	0.381	transcription factor, nucleic acid binding
KRT2	P35908	Keratin, type II cytoskeletal 2 epidermal	7.82	3	0.411	0.329	0.428	0.471	structural protein
SP100	P23497	Nuclear autoantigen Sp-100	9.56	6	0.412	0.438	0.352	0.363	HMG box transcription factor, signaling molecule
B2M	P61769	Beta-2-microglobulin	37.82	4	0.419	0.413	0.369	0.332	major histocompatibility complex antigen
ALB	A0A0C4DGB6	Serum albumin	16.89	9	0.427	0.455	0.668	0.654	transfer/carrier protein
BANF1	O75531	Barrier-to-autointegration factor	34.83	2	0.429	0.467	0.308	0.408	DNA binding, DNA integration
IFIT5	Q13325	Interferon-induced protein with tetratricopeptide repeats 5	19.71	7	0.438	0.497	0.477	0.435	RNA-binding
ERAP2	Q6P179	Endoplasmic reticulum aminopeptidase 2	6.25	5	0.461	0.527	0.371	0.457	metalloprotease
HLA-A	P01892	HLA class I histocompatibility antigen, A-2 alpha chain	64.38	15	0.465	0.509	0.415	0.447	immunoglobulin receptor superfamily
NDRG1	Q92597	Protein NDRG1	27.41	6	0.468	0.541	0.260	0.246	stress-responsive protein
STAT2	P52630	Signal transducer and activator of transcription 2	10.93	5	0.478	0.617	0.347	0.442	transcription factor, nucleic acid binding
HLA-E	P13747	HLA class I histocompatibility antigen, alpha chain E	24.02	2	0.479	0.319	0.472	0.433	immunoglobulin receptor superfamily
ATP6V0C	P27449	V-type proton ATPase 16 kDa proteolipid subunit	11.61	1	0.484	0.353	0.906	0.805	hydrolase, ATP synthase
UCHL3	P15374	Ubiquitin carboxyl-terminal hydrolase isozyme L3	15.65	2	0.492	0.459	0.558	0.220	cysteine protease
EPN2	F6PQP6	Epsin-2 (Fragment)	19.56	7	0.496	0.576	0.262	0.295	endocytosis
DBI	P07108	Acyl-CoA-binding protein	65.52	6	0.501	0.502	0.263	0.148	transfer/carrier protein
SP110	G5E9C0	SP110 nuclear body protein, isoform CRA_b	5.48	2	0.506	0.473	0.464	0.439	HMG box transcription factor, signaling molecule
TCEAL3	Q969E4	Transcription elongation factor A protein-like 3	16.50	2	0.507	0.478	0.354	0.307	transcription factor
LGALS3BP	Q08380	Galectin-3-binding protein	39.83	19	0.507	0.533	0.427	0.457	receptor, serine protease
UBE2L6	O14933	Ubiquitin/ISG15-conjugating enzyme E2 L6	59.48	5	0.517	0.407	0.382	0.317	ligase
SMYD2	Q9NRG4	*N*-lysine methyltransferase SMYD2	7.39	3	0.519	0.669	0.327	0.243	transcription cofactor
TREX1	Q9NSU2	Three-prime repair exonuclease 1	7.86	2	0.526	0.463	0.482	0.389	catalytic activityi
AK4	P27144	Adenylate kinase 4, mitochondrial	49.33	8	0.529	0.500	0.381	0.422	nucleotide kinase
FAM96B	J3KS95	Mitotic spindle-associated MMXD complex subunit MIP18 (Fragment)	23.58	2	0.539	0.421	0.452	0.473	iron-sulfur cluster assembly
DPP7	Q9UHL4	Dipeptidyl peptidase 2	35.37	12	0.541	0.581	0.365	0.431	serine protease
PML	P29590	Protein PML	33.79	22	0.545	0.558	0.424	0.392	activator
AGA	P20933	N(4)-(beta-*N*-acetylglucosaminyl)-l-asparaginase	24.86	5	0.551	0.635	0.415	0.490	protease
EPHA2	P29317	Ephrin type-A receptor 2	19.67	15	0.555	0.523	0.386	0.395	nervous system development
SERPINI1	Q99574	Neuroserpin	8.78	3	0.564	0.757	0.257	0.217	serine protease inhibitor
PAPSS2	O95340	Bifunctional 3′-phosphoadenosine 5′-phosphosulfate synthase 2	37.30	18	0.567	0.544	0.336	0.276	nucleotidyltransferase
IDUA	P35475	Alpha-l-iduronidase	31.85	16	0.572	0.605	0.416	0.479	glycosidase
GLA	P06280	Alpha-galactosidase A	25.64	8	0.574	0.625	0.464	0.435	glycosidase, hydrolase
SGSH	P51688	*N*-sulphoglucosamine sulphohydrolase	29.68	11	0.578	0.515	0.353	0.410	hydrolase
GAA	P10253	Lysosomal alpha-glucosidase	24.37	19	0.584	0.657	0.426	0.461	glucosidase
CHSY3	Q70JA7	Chondroitin sulfate synthase 3	6.92	6	0.587	0.483	0.361	0.320	glycosyltransferase
ACP5	K7EIP0	Tartrate-resistant acid phosphatase type 5 (Fragment)	36.54	1	0.587	0.544	0.313	0.246	glycosylated monomeric metalloprotein enzyme
PSMB8	P28062	Proteasome subunit beta type-8	39.13	8	0.590	0.552	0.455	0.493	endopeptidase activity
SPTBN2	O15020	Spectrin beta chain, non-erythrocytic 2	4.35	2	0.593	0.738	0.389	0.358	non-motor actin binding protein
PGM2L1	Q6PCE3	Glucose 1,6-bisphosphate synthase	48.07	30	0.598	0.627	0.492	0.448	glycosyltransferase, mutase
SAMD9L	Q8IVG5	Sterile alpha motif domain-containing protein 9-like	4.67	6	0.611	0.547	0.489	0.416	regulation of protein catabolic process
CSTB	P04080	Cystatin-B	45.92	3	0.615	0.680	0.328	0.380	cysteine protease inhibitor
LGMN	Q99538	Legumain	10.39	4	0.618	0.636	0.483	0.499	cysteine protease
CPQ	Q9Y646	Carboxypeptidase Q	20.55	7	0.620	0.631	0.406	0.452	carboxypeptidase activity
CTSA	P10619	Lysosomal protective protein	18.75	9	0.626	0.667	0.418	0.422	serine protease
NAGA	P17050	Alpha-*N*-acetylgalactosaminidase	11.92	3	0.626	0.648	0.480	0.287	deacetylase
ENO2	P09104	Gamma-enolase	60.83	11	0.631	0.676	0.436	0.494	lyase
GALNS	P34059	*N*-acetylgalactosamine-6-sulfatase	8.62	5	0.633	0.687	0.436	0.377	hydrolase
KCTD12	Q96CX2	BTB/POZ domain-containing protein KCTD12	33.23	11	0.634	0.624	0.446	0.480	enzyme modulator
GOLIM4	O00461	Golgi integral membrane protein 4	18.25	11	0.638	0.671	0.391	0.416	transport
NMRK1	B3KN26	Nicotinamide riboside kinase 1	12.26	1	0.641	0.527	0.422	0.402	kinase
RNASET2	D6REQ6	Ribonuclease T2	19.27	4	0.643	0.545	0.398	0.400	endoribonuclease activity
TUBB2B	Q9BVA1	Tubulin beta-2B chain	74.16	1	0.643	0.545	0.424	0.318	tubulin
MTAP	Q13126	*S*-methyl-5′-thioadenosine phosphorylase	71.38	15	0.645	0.683	0.484	0.492	phosphorylase
NAGLU	P54802	Alpha-*N*-acetylglucosaminidase	26.11	13	0.646	0.706	0.466	0.478	glycosidase, hydrolase
TXNIP	Q9H3M7	Thioredoxin-interacting protein	16.11	6	0.650	0.441	0.479	0.351	transcription regulation, oxidative stress mediator
BCAR3	O75815	Breast cancer anti-estrogen resistance protein 3	7.88	4	0.652	0.285	0.162	0.272	guanine-nucleotide releasing factor
GUSB	P08236	Beta-glucuronidase	26.42	16	0.678	0.649	0.495	0.452	galactosidase
PGK1	P00558	Phosphoglycerate kinase 1	84.41	31	0.686	0.647	0.461	0.437	carbohydrate kinase
H6PD	O95479	GDH/6PGL endoplasmic bifunctional protein	35.65	23	0.708	0.729	0.477	0.483	dehydrogenase
CSRP1	P21291	Cysteine and glycine-rich protein 1	64.25	9	0.711	0.670	0.426	0.427	actin family cytoskeletal protein
CPVL	Q9H3G5	Probable serine carboxypeptidase CPVL	21.22	9	0.711	0.640	0.480	0.453	serine protease
NNMT	P40261	Nicotinamide *N*-methyltransferase	56.06	10	0.713	0.667	0.335	0.333	methyltransferase
EXTL3	O43909	Exostosin-like 3	15.34	13	0.737	0.807	0.433	0.472	glycosyltransferase
VLDLR	P98155	Very low-density lipoprotein receptor	8.48	6	0.738	0.699	0.460	0.478	receptor, extracellular matrix glycoprotein
MMP14	P50281	Matrix metalloproteinase-14	19.76	11	0.748	0.800	0.344	0.361	hydrolase, metalloprotease, protease
OSTF1	Q92882	Osteoclast-stimulating factor 1	53.74	9	0.750	0.649	0.459	0.463	signal transduction
AKAP2	Q9Y2D5	A-kinase anchor protein 2	15.83	7	0.751	0.773	0.410	0.468	regulation of cell cycle, apoptosis process
SIAE	Q9HAT2	Sialate *O*-acetylesterase	12.05	5	0.769	0.725	0.346	0.294	esterase
MRC2	Q9UBG0	C-type mannose receptor 2	11.36	13	0.778	0.867	0.379	0.454	receptor
IDS	P22304	Iduronate 2-sulfatase	23.82	9	0.784	0.812	0.425	0.465	hydrolase
CNTNAP1	P78357	Contactin-associated protein 1	2.02	2	0.792	0.638	0.403	0.436	transporter, membrane-bound signaling molecule, receptor
AKR1C3	S4R3Z2	Aldo-keto reductase family 1 member C3	6.67	1	0.828	0.645	0.305	0.337	reductase
AMDHD2	Q9Y303	Putative *N*-acetylglucosamine-6-phosphate deacetylase	8.07	2	0.840	0.548	0.465	0.473	deacetylase
MANBA	O00462	Beta-mannosidase	6.60	3	0.901	0.799	0.356	0.482	galactosidase
SH3BP5L	Q7L8J4	SH3 domain-binding protein 5-like	5.09	2	0.912	0.931	0.354	0.411	protein kinase inhibitor
LRP1	Q07954	Prolow-density lipoprotein receptor-related protein 1	0.62	3	1.068	0.617	0.381	0.478	receptor, extracellular matrix glycoprotein
ATF7IP	F5GYR7	Activating transcription factor 7-interacting protein 1 (Fragment)	9.38	1	ND	0.447	0.441	0.146	transcription regulation
VPS29	Q9UBQ0	Vacuolar protein sorting-associated protein 29	56.04	1	ND	0.922	0.473	0.474	vesicle coat protein

^a^ The values of coverage and unique peptides are based on forward labeling result; ^b^ Ratios: Spot abundance of tachyplesin I-treated samples relative to the control; 0: control group; 10: 10 μg/mL dose group; 40: 40 μg/mL dose group; forward: forward labeling group; reverse: reverse labeling group; ^c^ Functional categories according to Gene ontology and panther biological process annotations.

**Table 3:** List of altered KEGG pathways with tachyplesin I treatment and their *p*-values identified by bioinformatic analysis using DAVID (*p* < 0.1).

Pathways	*p* Value	Differentially Expressed Proteins Involved in This Pathway
Lysosome	1.11 × 10^−8^	SGSH, AGA, NAGLU, GUSB, LGMN, ACP5, CTSA, MANBA, ATP6V0C, GLA, IDS, GALNS, NAGA, GAA, IDUA
Glycosaminoglycan degradation	2.53 × 10^−5^	SGSH, NAGLU, IDS, GUSB, GALNS, IDUA
Antigen processing and presentation	5.99 × 10^−4^	TAP2, LGMN, TAP1, HLA-A, HLA-B, HLA-E, TAPBP, B2M
DNA replication	3.51 × 10^−3^	MCM7, MCM2, MCM4, MCM5, MCM6
Type I diabetes mellitus	3.75 × 10^−2^	INS, HLA-A, HLA-B, HLA-E
Glycolysis/Gluconeogenesis	8.93 × 10^−2^	TPI1, ENO2, PGK1, ENO1

**Table 4:** Labeling scheme of DIGE for U251 gliomaspheres protein.

Gel No.	Cy2	Cy3	Cy5
Gel 01	Standard	A1	B2
Gel 02	Standard	B1	C3
Gel 03	Standard	C2	D3
Gel 04	Standard	D2	A2
Gel 05	Standard	A3	C1
Gel 06	Standard	B3	D1

A: control group; B: 10 μg/mL dose group; C: 40 μg/mL dose group; D: 80 μg/mL dose group; 1–3: three biological repeats in each group.

**Table 5:** Dimethyl-labeling scheme for U251 gliomaspheres protein.

Samples	Forward	Reverse
control group	Heavy (H)	Light (L)
10 μg/mL dose group	Light (L)	Medium (M)
40 μg/mL dose group	Medium (M)	Heavy (H)

## Supplementary Materials

The following are available online at www.mdpi.com/1660-3397/15/1/20/s1, Table S1: Total identified peptides information in the forward dimethyl labeling experiment, Table S2: Total identified peptides information in the reverse dimethyl labeling experiment, Table S3: Total identified proteins in the forward dimethyl labeling experiment, Table S4: Total identified proteins in the reverse dimethyl labeling experiment, Table S5: Transitions obtained in Parallel Reaction Monitoring (PRM).
